# Radio Frequency Fingerprint Identification for 5G Mobile Devices Using DCTF and Deep Learning

**DOI:** 10.3390/e26010038

**Published:** 2023-12-29

**Authors:** Hua Fu, Hao Dong, Jian Yin, Linning Peng

**Affiliations:** 1 School of Cyber Science and Engineering, Southeast University, Nanjing 210096, China; hdong@seu.edu.cn (H.D.); yjlaoshuaibi@163.com (J.Y.); pengln@seu.edu.cn (L.P.); 2 Purple Mountain Laboratories for Network and Communication Security, Nanjing 211111, China

**Keywords:** physical layer security, radio frequency fingerprint identification, 5G mobile device, PRACH preamble, differential constellation trace figure, convolutional neural network

## Abstract

The fifth-generation (5G) mobile cellular network is vulnerable to various security threats. Radio frequency fingerprint (RFF) identification is an emerging physical layer authentication technique which can be used to detect spoofing and distributed denial of service attacks. In this paper, the performance of RFF identification is studied for 5G mobile phones. The differential constellation trace figure (DCTF) is extracted from the physical random access channel (PRACH) preamble. When the database of all 64 PRACH preambles is available at the gNodeB (gNB), an index-based DCTF identification scheme is proposed, and the classification accuracy reaches 92.78% with a signal-to-noise ratio of 25 dB. Moreover, due to the randomness in the selection of preamble sequences in the random access procedure, when only a portion of the preamble sequences can be trained, a group-based DCTF identification scheme is proposed. The preamble sequences generated from the same root value are grouped together, and the untrained sequences can be identified based on the trained sequences within the same group. The classification accuracy of the group-based scheme is 89.59%. An experimental system has been set up using six 5G mobile phones of three models. The 5G gNB is implemented on the OpenAirInterface platform.

## 1. Introduction

The fifth generation (5G) of new radio (NR) has brought ultra-high-speed data transmission, extensive wireless coverage and low data latency. These advancements have driven the evolution of technologies such as the Internet of Things (IoT), autonomous driving and virtual reality (VR). However, the connectivity of massive devices makes network security even more critical, as 5G networks are vulnerable to physical layer threats including radio frequency (RF) jamming, spoofing and sniffing [1]. The claimed bit-level identities of a device (e.g., media access control (MAC) address, subscriber identity module (SIM) number and international mobile equipment identity (IMEI) number) can be easily spoofed [2] and are not secure enough for authentication. An effective device identification method is required to identify malicious attackers and enhance network security.

Radio frequency fingerprint (RFF)-based device identification is an emerging physical layer (PHY) authentication technique for wireless communication systems [3,4,5,6,7,8]. Due to the minor hardware manufacturing variations among electronic components, the transmitted signal waveform carries the inherent features of the device. These features are unique, persistent and resistant to cloning or tampering attempts, which can be considered the “fingerprint” of a device [2,9]. The research on RFF identification has covered a number of wireless communication systems, such as GSM [10], Wi-Fi [11,12,13,14,15], LoRa [16,17], ZigBee [2,18,19], RFID [20], LTE [21,22] and IEEE 802.11ad [23].

The source of RFF features includes crystal oscillator deviation [24,25], mixer imbalance [26,27], a transmission filter, amplifier nonlinearity [28,29] and antenna coupling. RFF features can be classified into two categories: transient features and steady state features. The transient features are extracted during the on-off transient period of the waveform, and the steady state features are extracted from the transmitted symbols. The steady state features include the carrier frequency offset (CFO) [25], in-phase/quadrature (I/Q) imbalance [26,27], sampling offset and amplifier nonlinearity [28,29].

With the application of machine learning algorithms in RFF identification, the overall signal can also be used as the RFF feature for classification. This approach can comprehensively consider the overall characteristics of the signal and preserve more signal details compared with the method of extracting a single feature. Raw I/Q samples with only CFO compensation were used in [30] for RFF identification of Zigbee’s device. The differential constellation trace figure (DCTF) was proposed in [18], where raw I/Q samples could be transformed into a stable two-dimensional DCTF even with the presence of a CFO. The DCTF provides a visualization of the statistical distribution of the I/Q samples after differential operation, which reflects the influence of the RFF features [18]. A convolutional neural network (CNN) is designed to identify the DCTF’s features.

The DCTF’s features have been studied with several kinds of wireless devices, exhibiting high identification accuracy. The performance of DCTF identification was studied using LoRa devices in [17]. The authors of [10] classified the DCTFs of global system for mobile communications (GSM) phones using a CNN. The DCTF of a long-term evolution (LTE) mobile terminal was studied in [21], where the DCTF of a physical random access channel (PRACH) preamble was identified. In this work, the DCTF is divided into transient parts and steady state parts, and then a multi-channel CNN is used with each channel treated a part of the DCTF.

In this paper, the DCTF features of a 5G mobile phone are studied based on the PRACH preamble. The PRACH preamble is the first signal transmitted by the user terminal for random access procedures. Device identification through PRACH signals can effectively prevent malicious devices from accessing the network and can also detect distributed denial of service (DDOS) attacks. Compared with the PRACH preamble of LTE, 5G NR supports more preamble formats. The formats A, B and C have been introduced to adapt to the flexible frame formats of NR. These preamble formats are much shorter than that of LTE. Moreover, multiple root values are required to generate the 64 preamble sequences of one cell, while for the long preamble of LTE, one root value is sufficient. With the study in this paper, we note that it is difficult to use a single CNN model to uniformly identify all the 64 preamble signals of formats A, B and C. Hence, an index-based DCTF identification scheme is proposed where the CNN model is trained for each PRACH preamble sequence. Moreover, for the case where the gNodeB (gNB) does not have the database of all the preamble sequences, the unknown sequence can also be identified through a *group*-based DCTF identification scheme. The transient features of a 5G mobile phone were studied in [31], but the identification accuracy was only 75.8% with four devices. The main contributions of this paper can be summarized as follows:

- An index-based DCTF identification scheme is proposed for the RFF identification of 5G mobile phones. The identification accuracy reaches 92.78% with six devices.
- When the database of all the preamble sequences is not available at the gNB, a *group*-based DCTF identification scheme is proposed to identify the unknown sequence based on known sequences within the same *group*, where preamble sequences generated from the same root value are grouped together. The classification accuracy of this case is 89.59%.
- An experimental system has been set up using 5G gNB and six 5G mobile phones of three models. The 5G gNB and core network are built with the OpenAirInterface (OAI) platform and universal software radio peripheral (USRP) B210. The PRACH signals are collected using a USRP N210.

Due to the frequency calibration between the mobile phone and the base station, CFO cannot be used as a standalone RFF feature for 5G phones. For comparison purposes, the frequency domain equalization (FDE) method [15], which consists of using the frequency domain-equalized I/Q samples with a rebuilt CFO, and an I/Q imbalance feature [27] are also simulated. The experimental results show that the index-based DCTF identification scheme outperformed the other methods for all the SNR values. The *group*-based DCTF identification scheme performed similarly to the FDE method at a low SNR, while at a high SNR, this scheme outperformed the FDE method.

The rest of this paper is organized as follows. In Section 2, the DCTF extraction method for a 5G PRACH preamble is introduced. Section 3 presents two CNN models for DCTF classification and proposes two identification schemes for two scenarios with different levels of knowledge of the preamble sequences. The experimental system and results are presented in Section 4. Section 5 concludes the paper.

## 2. RFF Extraction Based on a 5G NR PRACH Preamble

In this section, we first introduce the formula for the 5G PRACH preamble and then present the process of DCTF extraction based on the 5G PRACH preamble.

### 2.1. Formula for the 5G PRACH Preamble

In a 5G system, the gNB periodically broadcasts the synchronization block (SSB), which consists of the primary synchronization signal (PSS), secondary synchronization signal (SSS) and physical broadcast channel (PBCH). Mobile phone terminals initiate random access procedures with the gNB upon receiving the SSB. During this random access process, three primary uplink channels are used, such as the physical uplink control channel (PUCCH), physical uplink shared channel (PUSCH) and PRACH. The PUSCH and PUCCH are responsible for transmitting uplink data and uplink control signaling, respectively. However, due to variations in the frame format and frequency band, the PUSCH and PUCCH are less suitable for RF fingerprint extraction. On the other hand, the PRACH is used for uplink synchronization between the user equipment (UE) and the gNB. The PRACH signal is the first signal the UE sends to the gNB, characterized by a fixed bandwidth and frequency assigned by the base station. Due to the random access mechanism, each UE selects one of the 64 randomly assigned PRACH sequences. However, since the PRACH signal does not carry any identity information, the gNB cannot detect malicious access UE through the PRACH signal. This enables potential flooding attacks based on PRACH signals, and existing 3rd Generation Partnership Project (3GPP) specifications do not have any preventive measures against such attack methods [1].

**Figure 1:**
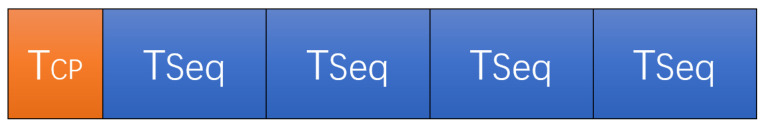
Structure of PRACH preamble format A2.

The PRACH preamble consists of a cyclic prefix (CP) and several repeated preamble sequences generated using the Zadoff–Chu (ZC) sequence. Figure 1 illustrates the time domain structure of the PRACH preamble format A2, where four ZC sequences are repeated in one frame.

**Figure 2:**
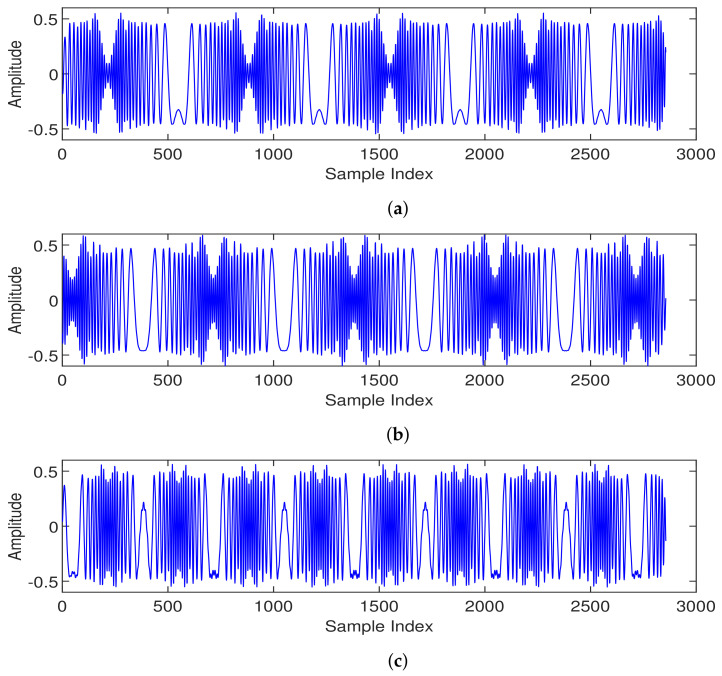
Different PRACH preambles of one cell (in-phase channel): (**a**) preamble index 1; (**b**) preamble index 2 and (**c**) preamble index 11.

The ZC sequence, a kind of constant-amplitude zero autocorrelation sequence (CAZAC), possesses desirable properties, such as a constant envelope and ideal periodic autocorrelation and cross-correlation characteristics [32,33]. Moreover, the Fourier transformation does not alter the characteristics of the ZC sequence. The ZC sequence can be generated using the following equation:

(1) $$x_{\mu }\left[i\right]=e^{-j\frac{\pi \mu i(i+1)}{L_{ZC}}},\;i=0,1,\cdots ,L_{ZC}-1$$

where $L_{ZC}$ is the length of the ZC sequence. Referring to 3GPP technical specification (TS) 38.211 in Table 6.3.3.1-1 and Table 6.3.3.1-2 in [34], we observe that 5G supports four types of long sequence preambles with a length of 839 and nine types of short sequence preambles with a length of 139. The high-level parameter “prach_RootSequenceIndex_PR” determines the specific length of the preamble sequence, where μ represents the root of the ZC sequence. Once the root sequence is generated as $\lfloor L_{ZC}/N_{CS}\rfloor -1$, other ZC sequences can be created by applying a cyclic shift to the root sequence, where ⌊·⌋ represents the floor value and $N_{CS}$ is the step size for the cyclic shift, while the value of $N_{CS}$ can be derived from Table 6.3.3.1-7 in TS 38.211 [34]. Hence, the $v^{th}$ sequence generated using the root value μ can be expressed as

(2) $$x_{u}^{v}\left[i\right]=x_{u}\left[\left(i+C_{v}\right)\;mod\;L_{ZC}\right],\;i=0,1,\cdots ,L_{ZC}-1$$

(3) $$C_{v}=vN_{CS},\;v=0,1,\cdots ,\left\lfloor L_{ZC}/N_{CS}\right\rfloor -1$$

where $C_{v}$ is called the cyclic shift value. Since each cell requires 64 preamble sequences, if the root sequence does not yield 64 ZC sequences (e.g., when $N_{CS}=34$ and $L_{ZC}=139$), then we have $\lfloor L_{ZC}/N_{CS}\rfloor =4$, indicating that only 4 ZC sequences can be generated based on one root value. In this case, 16 root values are required to generate all 64 preamble sequences. The first root is determined by the base station parameter “prach_RootSequenceIndex”, and the other roots are the natural numbers following the first root. We note that these root values are logical roots, and the corresponding physical roots can be found in Table 6.3.3.1-4 in TS 38.211 [34], denoted as μ in the formula. To launch the access requirement, the UE randomly selects one of the 64 preamble sequences. Examples of the different PRACH preambles for one cell are illustrated in Figure 2, where the periodic structure can be observed. Moreover, the preamble sequences generated from the same root value (i.e., Figure 2a,b) exhibited high similarity on the waveform.

In this paper, we use the term “*group*” to refer to the preamble sequences generated from the same root value. In our experiment, each *group* consisted of four preamble sequences (i.e., $\lfloor L_{ZC}/N_{CS}\rfloor =4$). Hence, a total of 16 *groups* were used to generate the PRACH preambles. Detailed gNB parameters are provided in Section 4.

### 2.2. Signal Preprocessing

**Figure 3:**
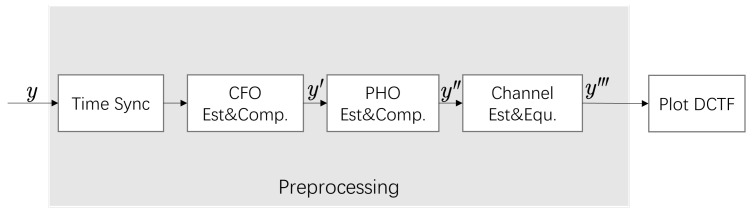
Signal preprocessing flowchart.

Signal preprocessing involves time synchronization, CFO and phase offset (PHO) estimation and compensation, channel estimation and channel equalization, as shown in Figure 3.

(1) ***Time synchronization***. The starting point of the received baseband signal *y* must be well located to extract the PRACH signal properly. In this paper, we determine the starting point through the repeatability of CP in the signal such that
  (4) $$D=\underset{i}{arg\max}\left\{\sum_{j=0}^{L_{CP}-1}y\left(i+j\right)y^{*}\left(i+j+L_{inter}\right)\right\},i=1,\cdots ,L$$
  where $L_{CP}$ and $L_{inter}$ represent the theoretical CP length and the signal length without CP, respectively, and *L* is the difference between the received signal length and the theoretical signal length.
(2) ***CFO estimation and compensation***. CFO is caused by the difference between oscillator frequencies at the transmitter and receiver. It is also estimated based on the CP of the PRACH signal such that
  (5) $$\Delta f=\frac{\sum_{k=1}^{L_{CP}}angle\left(y\left(k\right)y^{*}\left(k+L_{inter}\right)\right)}{2\pi L_{CP}L_{inter}}R_{s}$$
  where angle(·) refers to the phase angle and $R_{s}$ is the sampling frequency of the received signal. The CFO-compensated signal is given by
  (6) $$y^{\prime }\left(k\right)=y\left(k\right)e^{-\frac{j2\pi \Delta fk}{R_{S}}}.$$
(3) ***PHO estimation and compensation***. The phase offset can be estimated as follows:
  (7) $$\phi =angle(\frac{1}{L_{CP}+L_{inter}}\sum_{k=1}^{L_{CP}+L_{inter}}y\left(k\right)\cdot x_{l}^{*}\left(k\right))$$
  where $x_{l}\left(k\right)$ represents the corresponding standard PRACH signal. The PHO can be compensated by
  (8) $$y^{\prime \prime }\left(k\right)=y^{\prime }\left(k\right)e^{-j\phi }.$$
(4) ***Channel estimation and equalization***. In our experiment, the UE worked in the 5G NR n78 frequency band, and hence the PRACH signals were affected by multipath channel fading. Least squares (LS) channel estimation and zero forcing (ZF) equalization techniques were applied to the signal to mitigate the influence of channel fading. The channel estimation can be expressed as [15]
  (9) $$\hat{H}=\frac{y^{\prime \prime }}{x}$$
  where *x* is the standard transmit signal. In our study, we selected the first and last 200 samples of the signal for channel estimation.

The ZF equalization mitigated the channel distortion for RFF extraction. The equalized signal can be expressed as

(10) $$y^{\prime \prime \prime }=\frac{y^{\prime \prime }}{\hat{H}}.$$

**Figure 4:**
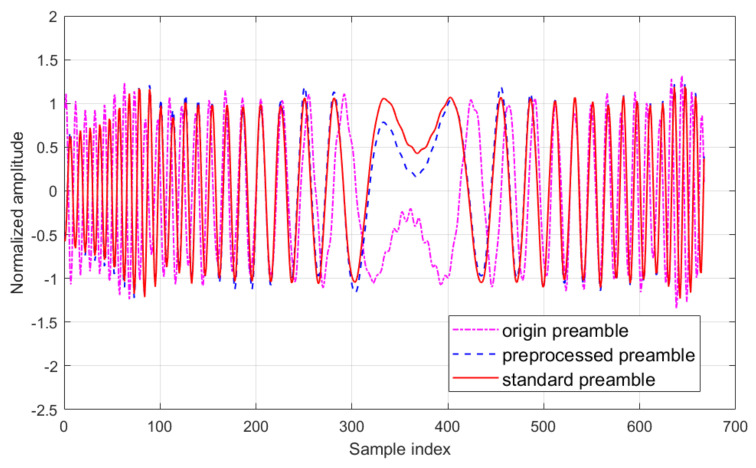
A segment of preamble waveform after preprocessing (in-phase channel).

A segment of the waveform after preprocessing is presented in Figure 4. The original received signal and the standard signal are also illustrated for comparison purposes. It can be observed that the preprocessed signal was quite similar to the standard signal.

**Figure 5:**
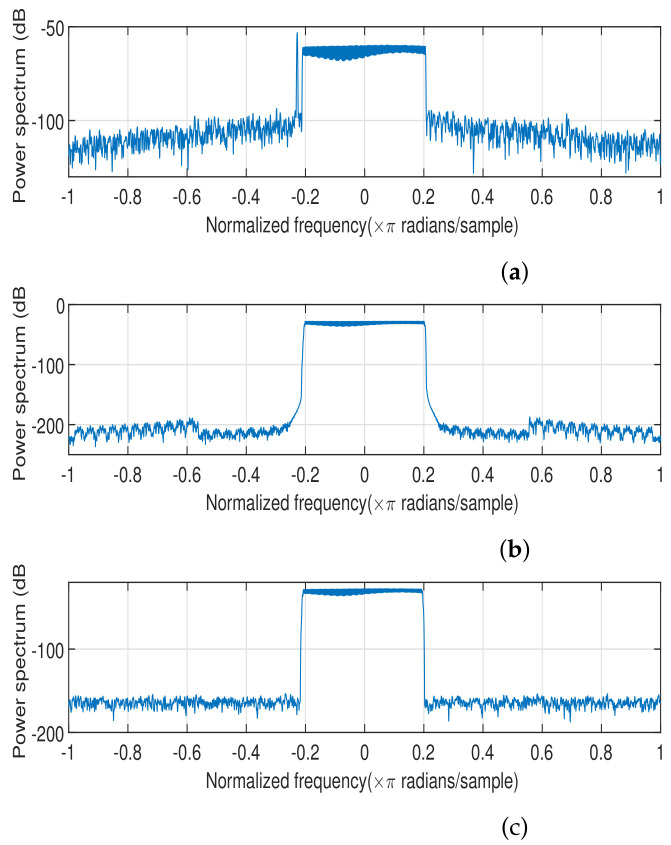
The power spectrum of (**a**) the collected original signal, (**b**) the standard signal and (**c**) the preprocessed signal.

The power spectra of the signals before and after preprocessing are illustrated in Figure 5. It can be observed that thanks to the signal preprocessing, especially the channel equalization, the frequency domain distortions were mitigated.

### 2.3. DCTF Extraction for the 5G PRACH Signal

For DCTF extraction, the signal power was rearranged such that

(11) $$\tilde{y}\left(k\right)=\frac{y^{\prime \prime \prime }\left(k\right)}{\frac{1}{L_{CP}+L_{inter}}\sum_{k=1}^{L_{CP}+L_{inter}}\left(\right|y^{\prime \prime \prime }\left(k\right)\left|\right)}\lambda$$

where |·| denotes the absolute value and λ represents the scaling parameter. In our experiment, the value of λ was 0.8, which has been verified to be suitable for a 65×65 DCTF feature size.

The DCTF figure can be obtained with the help of a differential operation [18], in which the elements are written as

(12) $$D\left(t\right)=\tilde{y}\left(t\right)\cdot {\tilde{y}}^{*}\left(t+t_{n}\right)$$

where ${\left(\cdot \right)}^{*}$ represents the conjugation and $t_{n}$ is the differential time interval. In our experiment, $t_{n}$ is one symbol of time. The differential operation enables the extraction of stable constellation features even when there are residual CFOs in the signal [18]. The measurement matrix Φ can be generated with a size of M×M pixel grids ranging from −A to *A*. The values of elements $\Phi_{j,k}$ were initialized as zero. For each D(t), we found the index (j,k) of Φ through the following process:

(13) $$j=\left[\frac{D_{I}\left(t\right)+A}{2A}M\right],\;k=\left[\frac{D_{Q}\left(t\right)+A}{2A}M\right]$$

where [·] is the round value and the values of *M* and *A* are fixed to 65 and 32, respectively. Here, $\Phi_{j,k}$ increases by one when D(t) falls to the index (j,k). Finally, when all of the samples are counted and added to the specific index of the measurement matrix, the distribution density of the entire waveform can be represented with Φ. The elements of Φ are normalized by rescaling the pixel values from 0 to 255.

**Figure 6:**
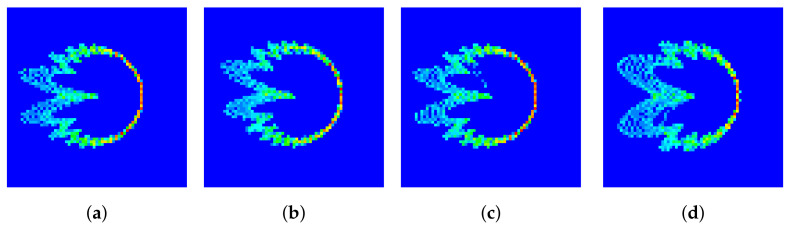
DCTF for (**a**) standard preamble with index 1, (**b**) preamble with index 1 of UE1, (**c**) standard preamble with index 2 and (**d**) standard preamble with index 11.

The DCTFs generated from the standard preamble sequence and received signal are illustrated in Figure 6a,b, respectively. The red points correspond to the positions with higher values. It can be seen that the two DCTF matrices were not exactly the same due to the thermal noise at reception and the RFF features of the device. Moreover, we note that the preambles generated using the same root value (i.e., Figure 6a,c) exhibited similar DCTF features, which were quite different from the DCTF features of the preamble generated from a different root value (i.e., Figure 6d). In the *group*-based DCTF identification scheme proposed in Section 3.3, the preambles generated using the same root value are grouped together.

## 3. RFF Identification Scheme

In the signal collection experiment conducted using real 5G mobile phones, the inherent randomness of the PRACH preamble sequence used by the mobile phones to access the gNB led to an inability to capture all 64 PRACH preambles for each phone. Consequently, we defined two scenarios based on the extent to which the gNB possessed knowledge of the PRACH preambles:

- Scenario 1: The gNB possesses complete knowledge of all the PRACH preambles used by the phone. In this scenario, the types of PRACH preamble sequences employed for training are the same as those used for testing during RFF identification.
- Scenario 2: The gNB has only partial knowledge of the PRACH preambles used by the phone. This implies that all the types of PRACH preamble sequences used for training become a subset of the PRACH preamble sequences employed for testing during RFF identification.

In this section, we first introduce the existing DCTF-based CNN structures (i.e., the single-channel CNN and multi-channel CNN). Then, an index-based DCTF identification scheme is proposed for Scenario 1 using a multi-channel CNN, and a *group*-based DCTF identification scheme is proposed for Scenario 2 using a single-channel CNN.

### 3.1. Existing DCTF-Based CNN Structures

Drawing from existing research on DCTFs in fields such as LTE, and considering the characteristics of the 5G PRACH preamble, two CNN structures were used to classify the DCTF of a 5G PRACH signal. The single-channel CNN structure synthesized the overall features, and the multi-channel CNN structure emphasized the transient part of the signal.

#### 3.1.1. Single-Channel DCTF-Based CNN Structure

**Figure 7:**
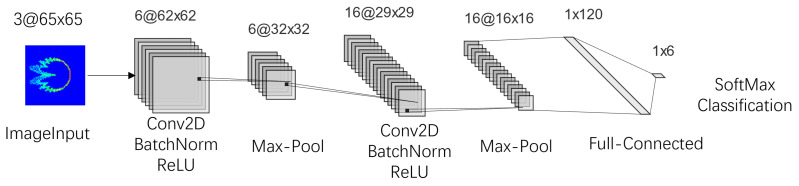
Single-channel DCTF-based CNN structure.

The DCTF extracted from the PRACH signal was fed into a CNN following the structure of LeNet-5 [35]. Originally proposed for high-performance handwritten digit identification, the application of LeNet-5 is similar to our experiment, where we needed to recognize six 5G mobile phones. Utilizing more complex network structures would significantly increase the computational complexity, with limited potential for performance improvement. The specific layers are illustrated in Figure 7, consisting of six layers. Convolutional layers capture the local features of the RFF, with the convolution kernel sizes set to [3×3]. Hidden layers following the convolutional layers included BatchNorm to enhance the training speed and generalization capability and ReLU to introduce nonlinearity and alleviate the vanishing gradient problem. Additionally, pooling layers were employed to reduce the size of the parameter matrices and the number of parameters in the fully connected layers, speed up computation and prevent overfitting. In this stage, max-pooling layers with a stride of 2 and kernel size of [2×2] were used.

#### 3.1.2. Multi-Channel DCTF-Based CNN Structure

**Figure 8:**
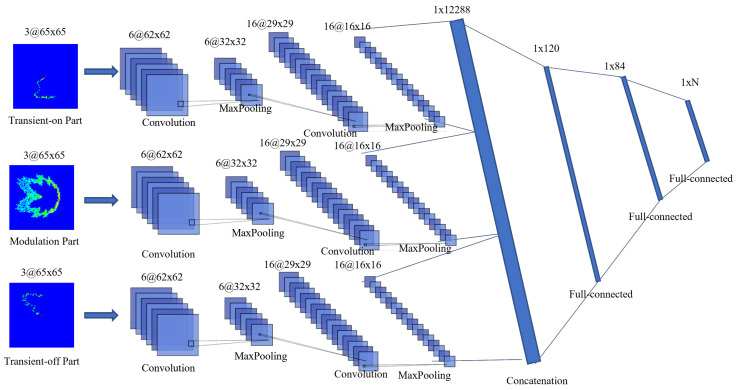
Multi-channel DCTF-based CNN structure.

In [21], the multi-channel DCTF-based CNN demonstrated outstanding performance for LTE terminals. This inspired us to apply it to the 5G terminals. The multi-channel approach consists of generating separate DCTFs from the transient on and off parts and the steady state part in order to enhance the recognition of transient features. Consequently, each signal was transferred to three DCTF features. As shown in Figure 8, these three DCTFs were the inputs of the multi-channel CNN. The components of each CNN channel were same as those in the single-channel CNN, and the outputs of the three channels were connected through the concatenation layer.

### 3.2. Index-Based DCTF Identification Scheme

An index-based DCTF identification scheme is proposed for Scenario 1 using a multi-channel CNN. The multi-channel CNN accentuated the device disparities by emphasizing the transient parts, which led to performance enhancement compared with the single-channel CNN. The experimental results are presented in Section 4. In the index-based DCTF identification scheme, because the complete knowledge of all the preamble sequences was available at the gNB, the CNN model was trained separately for each preamble sequence. Once the testing signal was received, the index of the preamble sequence could be estimated by computing the cross-correlation with the standard preamble sequences. Then, the extracted DCTFs would be entered into the corresponding CNN model for device classification.

### 3.3. Group-Based DCTF Identification Scheme

**Figure 9:**
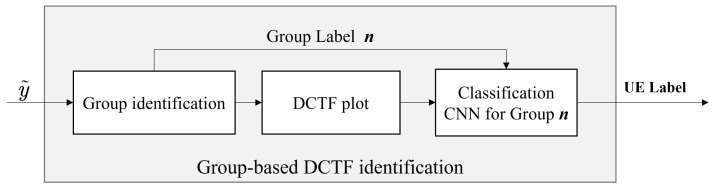
Group-based DCTF identification scheme using a single-channel CNN.

The randomness of PRACH sequence selection makes it difficult for the gNB to obtain sufficient learning samples for all the preamble sequences through long-term monitoring (i.e., the gNB has only partial knowledge of the PRACH preambles). In response to this problem, Scenario 2 studies the case where the PRACH preambles used for training are only a subset of the PRACH preambles used for testing (i.e., certain PRACH preambles received for testing have not been trained by the gNB). Based on the characteristics of the ZC sequences, we note that the PRACH preambles generated using the same root μ (i.e., the preamble sequences of the same *group*) were the cyclic shifted version of the same root sequence. As a result, DCTFs of the same *group* exhibited similarity. Hence, when one preamble sequence within a *group* was trained, other preamble sequences within the same *group* could also be classified based on similar features. Therefore, a group-based DCTF identification scheme is proposed for Scenario 2, as illustrated in Figure 9. The CNN model was trained separately for each *group*. During the testing stage, the group label was determined by the sequence index, and then the extracted DCTFs would be entered into the corresponding CNN model for device classification. Hence, the number of CNN models to be trained was same as the number of *groups*. The group-based DCTF identification scheme uses a single-channel CNN because a multi-channel CNN reduces the similarity among DCTFs within the same *group* by highlighting the transient parts, which weakens the differences among DCTFs between different groups.

## 4. Experimental Results

In this section, we first present the experimental set-up and signal collection environment. Then, the performance of the proposed DCTF identification schemes is evaluated.

### 4.1. Experimental Set-up and Data Collection

**Figure 10:**
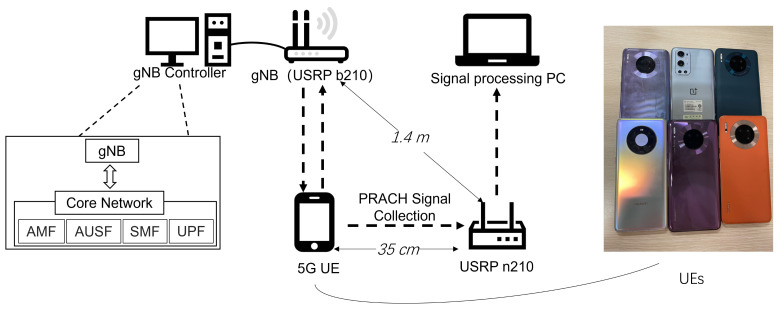
Experimental set-up and six 5G mobile phones.

**Figure 11:**
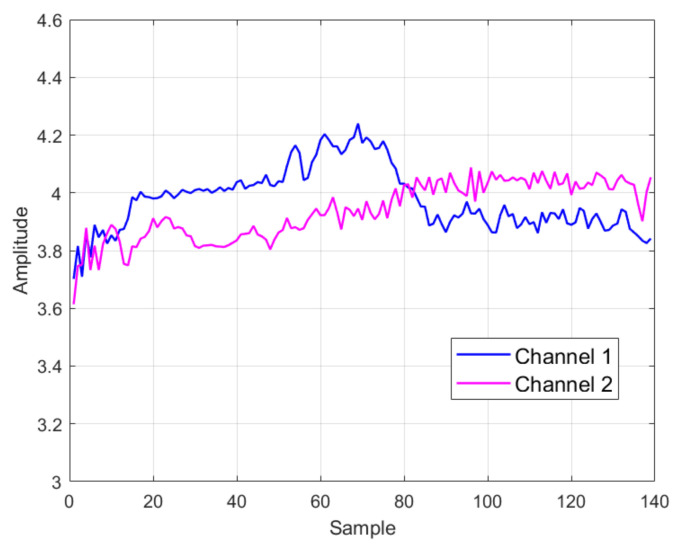
Examples of frequency domain channel fading for one PRACH preamble of one phone.

The experimental system included a gNB and 6 5G mobile phones, as illustrated in Figure 10. The gNB was built with a USRP B210 and the OAI platform [36,37], and the specific parameters are listed in Table 1. The 5G core network was also established using OAI, with Docker containers and Docker Compose to facilitate the installation and management. The models of the 6 mobile phones are described in Table 2, including 4 Huawei Mate30 phones, 1 Huawei Mate40 phones, and 1 OnePlus 9 Pro phone. A test subscriber identity module (SIM) white card was used with the mobile phones to enable access to the gNB. The transmitted PRACH signals were captured using the USRP N210 device with a sampling rate of 20 MHz. The positions of the USRP N210, gNB and the 6 mobile phones remained fixed during the PRACH signal capture process. The distance between the USRP N210 and the gNB was 1.4 m, and the distance between the mobile phones and the USRP N210 was 0.35 m. The measured signal-to-noise ratio (SNR) level was about 25 dB. We kept toggling the airplane mode of the phone on and off to force the phone to send PRACH signals. We could collect about 3000 signal samples per day. However, to obtain sufficient samples for each PRACH preamble from randomly transmitted PRACH signals, a total of at least 40,000 samples needed to be collected for each phone. The signal collection process extended for 3 months. Hence, the collected signal dataset included variations in both channel fading and time. Examples of the estimated frequency domain channel fading for one PRACH preamble of one phone are presented in Figure 11. It can be seen that the channel varied during the process of signal acquisition. This was due to the movement of people and changes in the surrounding objects. We note that real-world transmission scenarios are more complex, and we will test more scenarios, including non-line-of-sight (NLOS) channels and outdoor scenarios, in our future work.

**Figure 12:**
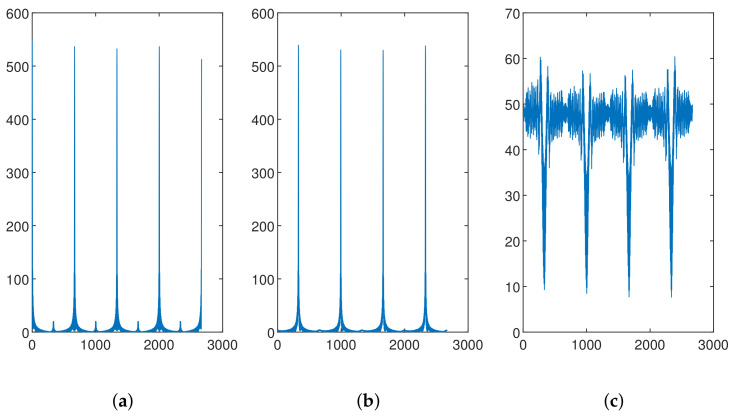
Cross-correlation between (**a**) preamble sequences of the same index, (**b**) preamble sequences from the same *group* and (**c**) preamble sequences from different *groups*.

After the signal was collected, it was necessary to determine which of the 64 preamble sequences was received. Hence, the cross-correlations between the received preamble and the 64 standard preambles were computed such that

(14) $$R_{x}y=\left|\mathrm{IFFT}\left(Y\left(f\right)X_{l}^{*}\left(f\right)\right)\right|,l=0,\cdots ,63$$

where Y(f) and $X_{l}\left(f\right)$ are the Fourier transform of the received preamble after time synchronization and the *l*th standard preamble, respectively, while IFFT(·) is the inverse fast Fourier transform. Examples of the cross-correlation results are presented in Figure 12. When the cross-correlation was computed based on the same preamble sequence, several significant peaks could be observed, and there was one peak at the beginning. When the cross-correlation was computed using preamble sequences of the same *group*, the peaks could be observed, but there was no peak at the beginning. When the cross-correlation was computed using preamble sequences from different *groups*, no peak could be observed.

More than 40,000 PRACH signals were collected for each mobile phone. However, 10 types of preamble sequences were never observed, although the mobile devices were supposed to randomly select the preamble sequences for the access request. To ensure the training requirements of the CNN, the data consisted of 27 types of preamble sequences used for training, which almost covered all the possible μ values. The number of samples collected for each sequence ranged from 1200 to 2000. To enhance the efficiency of the training process, a GTX 3060 GPU (Nvidia, Santa Clara, CA, USA) was utilized.

### 4.2. Performance of the Index-Based DCTF Identification Scheme

The index-based DCTF identification scheme was tested for Scenario 1. The CNN model was trained for each type of sequence among the 27 preamble sequences. In order to simulate the case where complete knowledge of all PRACH signals was available at the gNB, the testing set was established using the same 27 preamble sequences. The collected signal samples were randomly assigned into the training and testing sets at a ratio of 8:2. The data in the training and testing sets had no intersection.

**Figure 13:**
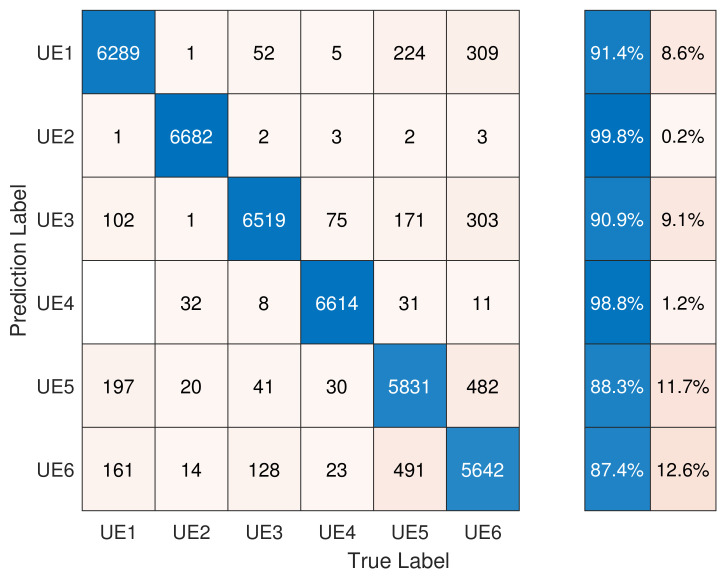
Performance of index-based DCTF identification scheme using a multi-channel CNN.

The classification results are presented in Figure 13. UE2 and UE4 showed an accuracy of around 99%. The high accuracy of these two devices was due to the fact that their models were different from the others. On the other hand, the other four devices belonged to the same model, and thus their RFF features were quite similar, which resulted in a lower classification accuracy. In conclusion, when the gNB had complete knowledge of the PRACH preamble sequences, the classification accuracy for the six devices was 92.78% using a multi-channel CNN.

**Figure 14:**
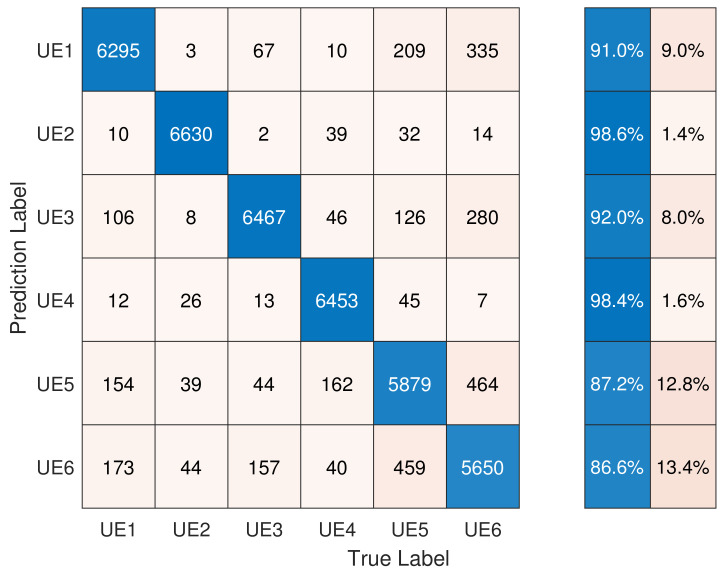
Performance of index-based DCTF identification scheme using a single-channel CNN.

Additionally, the performance of the single-channel CNN was also tested, and the results are presented in Figure 14. It can be observed that the classification accuracy of five devices slightly decreased, and the overall accuracy was 92.28%. This result demonstrates the advantages of the multi-channel CNN for Scenario 1, which emphasizes the transient features.

### 4.3. Performance of the Group-Based DCTF Identification Scheme

For Scenario 2, the gNB had only partial knowledge of the PRACH preambles used by the phone. Hence, the training set was composed of 27 types of preamble sequences, similar to Scenario 1, and the testing set was composed of 50 types of preamble sequences. The indexes of the sequences used for training and testing are listed in Table 3. The training set of Scenario 2 was the same as that in Scenario 1. The number of samples for each type of sequence of each device was fixed to 142 in the testing set. The CNN model was trained and tested using the sequences from the same *group*.

**Figure 15:**
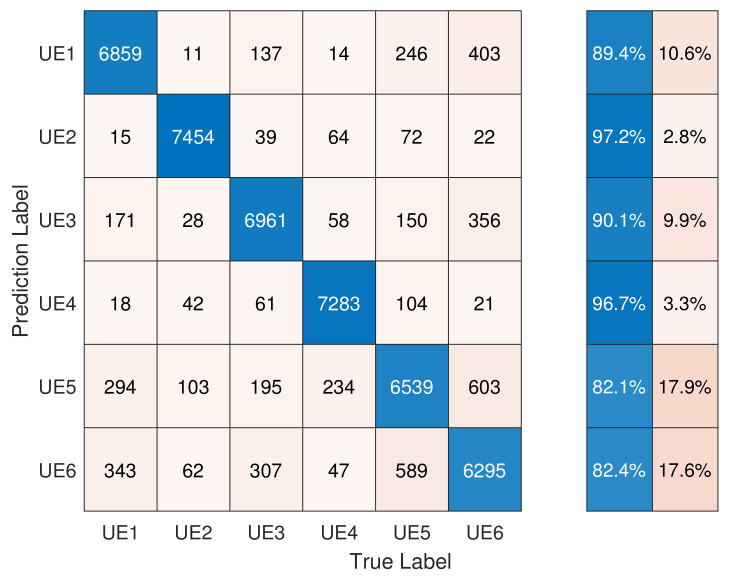
Performance of *group*-based DCTF identification scheme using a single-channel CNN.

The performance of the group-based DCTF identification scheme using a single-channel CNN is presented in Figure 15. We note that the classification accuracy of each device was slightly lower than that of Scenario 1. However, the accuracies of UE2 and UE4 both exceed 96%, which demonstrates that the RFF features of different types of devices were more distinguishable. The overall classification accuracy was 89.59%.

Moreover, the relevance between different types of preamble sequences within the same *group* was tested. Table 4 presents the classification results when using one type of preamble sequence for training and another type of preamble sequence belonging to the same group for testing. The single-channel CNN model was used. The results show that the identification accuracy depended on the spacing of preamble indexes. The adjacent indexes led to higher accuracy, while the indexes with a difference of three resulted in lower accuracy. Hence, it can be concluded that when the indexes between sequences within the same *group* were closer, their DCTF features were more similar.

**Figure 16:**
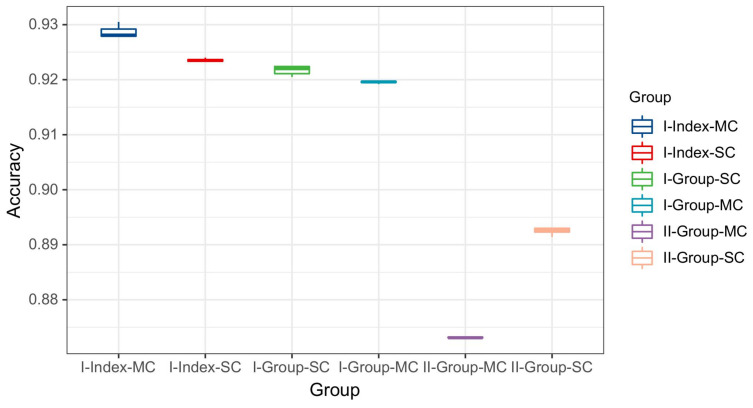
Classification results of different methods in Scenario 1 and Scenario 2.

Finally, to summarize the performance of the two proposed RFF identification schemes and clarify the appropriate CNN model for each scenario, the overall classification results are presented in Figure 16. To ensure the robustness of the results, a box plot with 15 repetitions has been used. In each repetition, the training and testing sets were reformed from the collected data samples with the help of a normally distributed random generator. For Scenario 1, four identification methods were compared: the index-based single-channel, index-based multi-channel, *group*-based single-channel and *group*-based multi-channel schemes. We note that the index-based multi-channel method outperformed the other methods in Scenario 1. However, the performance differences among the four methods were within 1%. For the *group*-based approach, the number of CNN models used could be significantly reduced. For Scenario 2, with the *group*-based approach, we found that the single-channel CNN outperformed the multi-channel CNN because the stability of the transient features within the *group* was not as good as that of the steady state features. Hence, emphasizing transient features leads to a degradation in classification performance.

### 4.4. Robustness and Sensitivity Analysis

**Figure 17:**
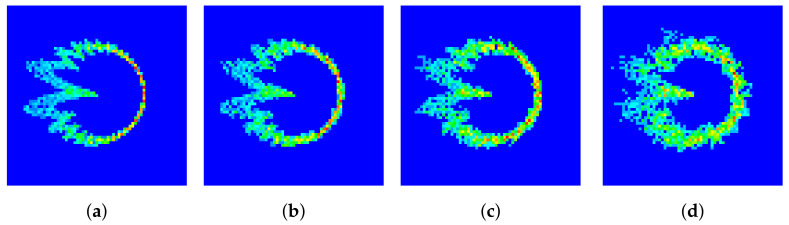
DCTF for PRACH preamble of index 1 with SNR of (**a**) 25 dB, (**b**) 20 dB, (**c**) 15 dB and (**d**) 10 dB.

**Figure 18:**
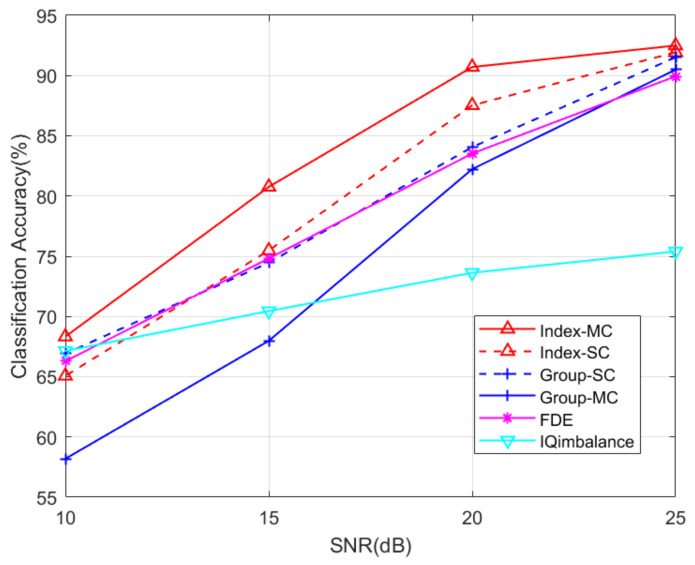
Classification results of different methods in function of SNR for Scenario 1.

**Figure 19:**
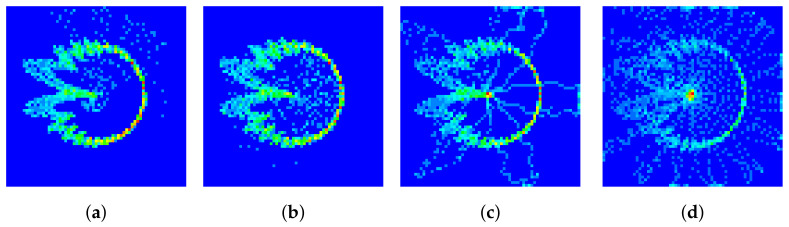
DCTF of UE1 for PRACH preamble of index 1 with an overlap ratio of (**a**) 1:16, (**b**) 1:8, (**c**) 1:4 and (**d**) 1:2.

In this part, the robustness and sensitivity of the proposed schemes are analyzed, and the impact of interference from other devices is also discussed:

(1) **Robustness.** The measured SNR of our experimental system was about 25 dB. To test the robustness of the proposed schemes in a low-SNR regime, we added Gaussian noise to the collected signals. The SNR levels were adjusted to 10 dB, 15 dB and 20 dB. The corresponding DCTFs are presented in Figure 17, where the impact of the noise can be observed. The classification results are presented in Figure 18.
(2) **Sensitivity.** The impact of the proportion of training and testing sets on the classification performance was tested, and the results are shown in Table 5. The training time is also presented. Taking into account both the training time and classification performance, we found that the proposed scheme was more efficient when the training and testing sets were at an 8:2 ratio.
(3) **Impact of interference.** Interference from other devices had a significant impact on the performance of RFF identification. We simulated the scenario where the transmissions of two devices overlapped. Assuming that UE1 and UE2 both transmitted the PRACH preamble of index 1, the DCTFs of UE1 with different overlap ratios are presented in Figure 19, where the head of the signal of UE2 is superimposed onto the tail of the signal of UE1. It can be seen from Figure 19 that as the overlap ratio increased, the deformation of the DCTF became significant.

The FDE method [15], which consists of using frequency domain-equalized I/Q samples with a rebuilt CFO, and the I/Q imbalance feature [27] were also simulated for comparison purposes. The I/Q samples of the FDE method are classified with a deep CNN [15]. The I/Q imbalance feature is extracted through a low-complexity algorithm that uses gain imbalance and phase imbalance as the indices of I/Q imbalance [27]. The I/Q imbalance features were classified using the k-nearest neighbors (KNN) algorithm.

It can be seen from Figure 18 that the index-based multi-channel scheme outperformed the other methods for all the SNR values. The performance of the *group*-based single-channel scheme was similar to that of the FDE method at a low SNR. However, this scheme showed an accuracy higher than that of the FDE method in a high SNR regime. The experimental results in Figure 18 demonstrate the robustness of the proposed schemes.

**Figure 20:**
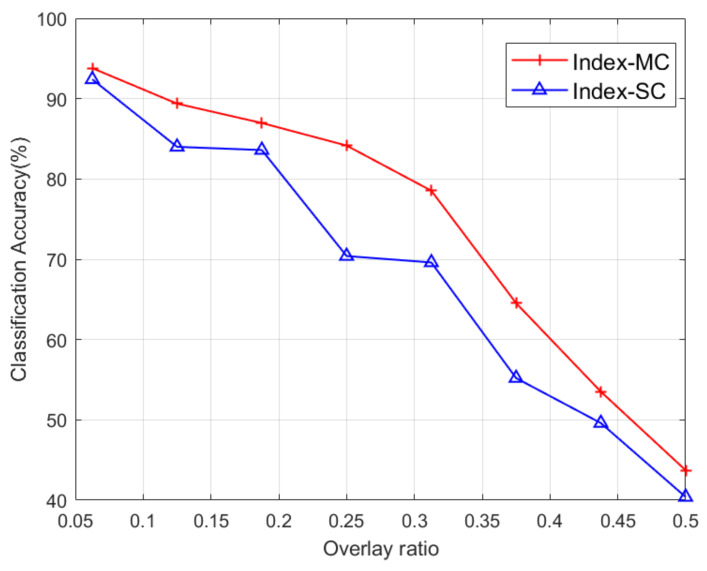
Classification results of UE1 as a function of the overlap ratio.

The classification performance of the overlapped signal is presented in Figure 20. It can be seen that as the overlap ratio increased, the classification accuracy decreased. However, the overlap case can be detected by computing the signal-to-interference ratio of the received signal.

## 5. Conclusions

In this paper, the performance of RFF identification was studied for 5G mobile phones. The DCTF features were extracted from PRACH preambles. Because the PRACH preambles of formats A, B and C were generated from multiple root values, an index-based DCTF identification scheme was proposed for the case where the database of all 64 PRACH preambles was available at the gNB. The classification accuracy of this scheme reached 92.78% with a multi-channel CNN. When only a portion of the preamble sequences could be trained at the gNB, a *group*-based DCTF identification scheme was proposed, where the untrained sequences could also be identified based on the CNN of trained sequences within the same *group*. The preamble sequences generated from the same root value belonged to the same *group*. The classification accuracy of this scheme was 89.59% with a single-channel CNN. We found that the stability of the transient features within the same *group* was not as good as that of the steady state features. To verify the performance of the proposed DCTF identification schemes, an experimental system was set up using a gNB platform and six 5G mobile phones, where the SNR of the system was 25 dB.

## Figures and Tables

**Table 1:** The gNB critical parameters.

Parameter	Value	Description
**frequencyBand**	78	Signals operating in the n78 frequency band
**subcarrierSpacing**	1	Subcarrier spacing is 30 kHz
**carrierBandwidth**	106	Carrier bandwidth is 106 resource blocks
**prach_ConfigurationIndex**	98	The preamble format is A2, and the length of the ZC sequence is 139
**zeroCorrelationZoneConfig**	13	The value of $N_{CS}$ is 34, and every ⌊139/34⌋=4 preamble sequences share a root value μ

**Table 2:** Mobile phone information.

Label	Model	CPU
UE1	Huawei Mate30	Kirin 990 5G
UE2	Huawei Mate40	Kirin 9000E
UE3	Huawei Mate30	Kirin 990 5G
UE4	OnePlus 9 pro	Qualcomm Snapdragon 888
UE5	Huawei Mate30	Kirin 990 5G
UE6	Huawei Mate30	Kirin 990 5G

**Table 3:** The indexes of sequences used for training and testing sets in Scenario 2.

Set	Index
Train	1 2 4 5 6 9 10 13 14 15 21 25 26 31 32 35 36 38 39 41 42 46 48 52 53 55 57
Test	1 2 3 4 5 6 8 9 10 12 13 14 15 16 21 22 23 25 26 29 30 31 32 33 34 35 36
	37 38 39 40 41 42 43 44 45 46 47 48 49 50 51 52 53 54 55 56 57 58 59 60

**Table 4:** Classification results with preamble sequences within the same *group*.

Cases	Train Preamble	Test Preamble	Group	Classification Accuracy
Adjacent indexes	P1	P2	[P1,P2,P3,P4]	92.08%
	P13	P14	[P13,P14,P15,P16]	91.54%
	P41	P42	[P41,P42,P43,P44]	89.80%
	P53	P54	[P53,P54,P55,P56]	93.07%
Indexes with a difference of 2	P1	P3	[P1,P2,P3,P4]	89.84%
	P13	P15	[P13,P14,P15,P16]	89.09%
	P41	P43	[P41,P42,P43,P44]	86.44%
	P53	P55	[P53,P54,P55,P56]	90.14%
Indexes with a difference of 3	P1	P4	[P1,P2,P3,P4]	87.39%
	P13	P16	[P13,P14,P15,P16]	87.56%
	P41	P44	[P41,P42,P43,P44]	84.16%
	P53	P56	[P53,P54,P55,P56]	86.74%

**Table 5:** Classification performance with different proportions of training and testing sets.

Proportion of Training and Testing Sets		6:4	7:3	8:2	9:1
Index-based DCTF-MC	Training time	413 s	465 s	524 s	571 s
	Accuracy	89.38%	90.83%	92.51%	92.45%
Index-based DCTF-SC	Training time	992 s	1164 s	1362 s	1663 s
	Accuracy	88.12%	90.14%	92.19%	92.25%

## Data Availability

The data presented in this study are available on request from the corresponding author.
